# Pericardial Fenestration With Monitored Anesthesia Care Using Diluted Remifentanil: A Series of 10 Cases

**DOI:** 10.7759/cureus.73054

**Published:** 2024-11-05

**Authors:** Tokimitsu Hibino, Yusuke Okui, Satoko Kondo, Fumiko Ogura, Yoshie Toba

**Affiliations:** 1 Department of Anaesthesiology, Seirei Hamamatsu General Hospital, Hamamatsu, JPN

**Keywords:** diluted remifentanil, monitored anesthesia care, pericardial fenestration, postoperative pericardial effusion, syringe pump

## Abstract

Introduction

Monitored anesthesia care (MAC) is an anesthesia management method in which anesthesiologists use the minimum necessary intravenous anesthetics to achieve sedation and pain relief while maintaining spontaneous breathing. The challenge for the anesthesiologist is to find the correct balance between inhibiting the patient’s stress response to invasive treatment, maintaining intraoperative hemodynamic stability, and doing so as quickly as possible. We hypothesized that diluting remifentanil (D-remi) to 10 μg/mL and increasing the rate of administration would increase the responsiveness of the syringe pump and allow for better control of anesthetic depth. In this study, we aimed to evaluate the efficacy of MAC with D-remi in critically ill patients.

Materials

We investigated 10 cases in which anesthesia was managed by MAC with D-remi during emergency pericardial fenestration after cardiovascular surgery at our institution. Each parameter related to the patients' background and circulatory dynamics was extracted retrospectively from the electronic medical records and anesthesia records. The target depth of anesthesia was set at Richmond Agitation-Sedation Scale (RASS) -3 to -4 on MAC with spontaneous breathing. We increased the infusion rate of this analgesic during more invasive procedures.

Results

In all cases, the emergency pericardial fenestration could be performed with stable hemodynamics throughout the procedure. Spontaneous respiration never disappeared in any case. Although there were six cases in which airway maintenance by mandibular elevation was required due to glossoptosis, the minimum intraoperative SpO_2_ was as high as 94% (92-100%), and none of the cases dropped below 92%.

Conclusions

The improved responsiveness of syringe pumps through the use of D-remi may be advantageous in situations where the infusion rate of anesthetic needs to be adjusted frequently and quickly. Since this is a small case study, we view our results cautiously and hope that further investigation of the usefulness of D-remi in MAC, including large-scale randomized studies, will be conducted in the future.

## Introduction

Monitored anesthesia care (MAC) is a continuous and broad concept that ranges between sedation and general anesthesia [1,2]. In this article, MAC is defined as a method of anesthesia management in which anesthesiologists use the minimum necessary intravenous anesthetics and analgesics to maintain spontaneous breathing and provide adequate pain relief for the patient. MAC is used for a variety of purposes; for example, better analgesia and sedation in surgeries performed under local anesthesia, immobilization in MRI scans of pediatric patients, and anesthetic management of critically ill patients for whom general anesthesia is itself a high-risk procedure [3]. The anesthesiologist must provide the patient with good analgesia and sedation so that the operation or examination can be performed safely. However, the degree of difficulty in achieving this goal varies greatly depending on the clinical situation in which MAC is performed.

The use of MAC in transcatheter aortic valve implantation (TAVI) for severe cases has emerged as a common alternative to general anesthesia, and studies showing MAC as the superior anesthetic management for TAVI have been widely documented. Dehédin et al., in a study comparing anesthetic management methods in TAVI, used continuous intravenous propofol and target-controlled infusion (TCI) of remifentanil as anesthesia methods in a local/regional anesthesia (LRA) or MAC group [4]. They adjusted the remifentanil target concentration (1 ng/mL to 3 ng/mL) to achieve a Ramsay score of 2-3. In applying MAC, or the aforementioned LRA, the basic strategy is to use shallow anesthesia as a base, increase the dose of anesthetics or opiates just before a strong invasion, and safely decrease the dose when possible. However, this approach of increasing or decreasing the anesthetic infusion rate is not feasible with agents that have a long half-life, such as fentanyl. Using agents with short half-lives, such as remifentanil, makes it easier to control the appropriate level of anesthesia according to the degree of invasiveness, but the anesthesiologist must be wary of administering an anesthetic concentration that can disturb spontaneous respiration.

We hypothesized that a high infusion rate of remifentanil prepared at a low concentration would enable us to adjust to the patient’s condition more quickly than the common method of administering a standard concentration at a low infusion rate. This hypothesis was based on the fact that there is a delay before the set infusion rate of the syringe pump is reached; the higher the set infusion rate, the smaller the delay [5,6], and vice versa. According to Yoshida et al., it takes about 30 minutes to reach the intended infusion rate using a low infusion rate and only five minutes using a high infusion rate [7]. Of course, using a normal concentration of opiates at a high infusion rate could cause loss of spontaneous respiration. Therefore, a diluted concentration of remifentanil at a higher infusion rate would allow the anesthesiologist to provide adequate stress inhibition with a lower risk of intraoperative hemodynamic instability or weakened respiration. Based on this fact, we decided to dilute remifentanil (D-remi) to 10 μg/mL and administer it at a high infusion rate. Thus, the purpose of this study was to investigate whether D-remi could be used to perform MAC safely in critically ill patients.

Emergency pericardial fenestration is an emergency procedure performed for circulatory compromise due to acute pericardial effusion. Pericardial effusions are a potential complication after cardiac surgery, with cardiac tamponade, characterized by Beck's triad, being a potentially lethal condition [8]. If patients undergoing emergency pericardial fenestration are given general anesthesia, they can suffer from severe tachycardia, hypotension, and even cardiac arrest [9]. These patients are some of the most vulnerable to anesthesia. Although MAC has been applied in various surgeries and clinical examinations at our institution, we decided to examine MAC performed for emergency pericardial fenestration after cardiovascular surgery in this study to illustrate that MAC can be performed safely for even the most critically ill patients. There were also practical reasons for our decision. Although general anesthesia may be used for pericardial fenestration [10], MAC is preferable, as a lower dose of anesthetic is less risky for the patient’s circulation. MAC performed while preserving spontaneous breathing is more useful in the anesthetic management of cardiac tamponade than general anesthesia for hemodynamic stability [11,12]. Inhalation associated with spontaneous breathing, which can reduce intrathoracic pressure and draw blood into the thorax, is important for maintaining venous return under cardiac tamponade [13], and deep inhalation allows patients to maintain a constant level of systemic venous return [14]. Additionally, emergency pericardial fenestration is a relatively simple procedure, and the routine procedure tends to proceed similarly in all cases, making it easier for the anesthesiologist to predict when the invasiveness of the procedure will become more severe and to adjust the amount of anesthesia. We retrospectively reviewed 10 patients who underwent emergency pericardial fenestration under MAC with D-remi.

## Materials and methods

Existing anesthesia and medical records were retrospectively reviewed and examined. Approval to conduct this study was obtained from the Institutional Review Board (IRB) of Seirei Hamamatsu General Hospital, Hamamatsu, Japan (IRB approval number: E-4446). This study was an observational study, and the IRB approved the opt-out disclosure of information about each patient's participation in the study. Opt-out information was posted on the hospital website.

The purpose of this study was to determine whether remifentanil can be used in a more dilute form than usual to safely perform MAC in critically ill patients. We investigated 10 cases in which anesthesia was managed by MAC with D-remi during emergency pericardial fenestration after cardiovascular surgery at Seirei Hamamatsu General Hospital between December 2014 and December 2020. The following data were extracted from the anesthesia and medical records of the 10 cases: minimum systolic blood pressure from induction to pericardial fenestration; maximum intraoperative noradrenaline rate; total noradrenaline dose; remifentanil dosing rate (at induction, before skin incision, before applying a chest opener, during intrapericardial aspiration, at the removal of the chest opener, and at the end of surgery); and total opioid dose (the potencies of remifentanil and fentanyl were considered to be the same, while the potency of morphine was assumed to be 1/50 of fentanyl). Patient background factors included age, sex, height, weight, previous medical history (hypertension, coronary artery disease, glucose intolerance, anticoagulant use, and antiplatelet use), American Society of Anesthesiologists-Physical Status (ASA-PS), and New York Heart Association (NYHA) cardiac function classification upon entering the operating room. Surgical factors included anesthesia method, airway maintenance method, induction medications, maintenance medications, presence of regional anesthesia, number of days since prior surgery, prior surgery classification (aorta/valvular/ischemic heart disease/adult congenital heart disease), duration of surgery, duration of anesthesia, lowest intraoperative percutaneous oxygen saturation (SpO_2_), intraoperative fluid infusion volume, intraoperative blood transfusion volume, intraoperative blood loss, intraoperative urine output, postoperative hypoxia (<90%), intensive care unit (ICU) length of stay, and one-year postoperative survival rate.

Management of anesthesia was performed using the following methods. The target depth of anesthesia was set at Richmond Agitation-Sedation Scale (RASS) -3 to -4 on MAC with spontaneous breathing. A face mask was attached to the respiratory circuit of the anesthesia machine, and oxygen was administered at 5 L/min. In all 10 cases, induction medications were dexmedetomidine, midazolam, and D-remi. Fentanyl, noradrenaline, and ketamine were added if deemed necessary by the anesthesiologist in charge. The same medications were used for maintenance as for induction, and morphine was used in one case. The anesthetic doses for each patient are shown in Table 1. Anesthesia was deepened if spontaneous eye opening, vocalization, or body movements were present. A sampling tube was placed near the patient's mouth to get a capnometer waveform, which was compared to the waveform before sedation. When the height of the waveform diminished, glossoptosis was suspected, and the airway was manually secured. If the waveform did not improve, anesthesia was decreased. Dexmedetomidine was the main sedative, with midazolam and ketamine as adjunctive agents. In one case, sevoflurane was used temporarily at the decision of the anesthesiologist in charge of intraoperative body movements. The respiratory rate was assessed visually and with a capnometer, and for analgesics, a small dose of D-remi was administered to keep the respiratory rate around 11 breaths/minute before the invasion. The surgical procedures performed in this study were similar, and the timing of increased invasiveness was predictable in all cases. Remifentanil was administered at increasingly higher dosage infusion rates at each of the following points: just prior to incision before the chest opener was applied, and just prior to aspiration of the pericardial sac. As the operation progressed to the closed wound phase and the treatment became progressively less invasive, the infusion rate of remifentanil was decreased to keep the respiratory rate at 11 breaths/minute. Remifentanil was administered in the range of 0.02 to 0.1 μg/kg/min but was administered beyond the pre-determined set range in one patient (0.21 μg/kg/min) who was considered to be in pain due to body movement and grimace. For an immediate increase in opiate blood concentration, a bolus dose of fentanyl was administered, and the remifentanil infusion rate was also increased. In each case, the rate of D-remi administration was recorded at each of the following timings: induction of anesthesia, skin incision, chest opener placement, aspiration of the pericardial sac, chest opener removal, and end of surgery. Rather than attempting to obtain the necessary analgesia with a single analgesic agent, multimodal analgesia, in which multiple analgesics with different mechanisms are used, is now recommended for perioperative pain management [15,16]. In our cases, we combined intravenous analgesia with regional anesthesia. Intercostal nerve blocks were performed if the patient's condition permitted, and local anesthesia in the operative field was also used. Because of the smooth induction of anesthesia and rapid pericardial fenestration, one anesthesiologist started the induction, and the other performed the intercostal nerve block. The patient was asleep when the intercostal nerve block was completed, so the effect of the intercostal nerve block could not be evaluated. If there was no time to perform a nerve block, only local anesthesia in the operative field was performed (Table 1).

**Table 1:** Anesthesia method and anesthetic dosage for each case The height and weight of each case, the dose of anesthetic, the rate of noradrenaline administration, and the method of regional anesthesia are shown. Dex: dexmedetomidine (μg/kg/h); MDZ: midazolam; R: remifentanil (diluted to 10 μg/mL) (μg/kg/min); F: fentanyl; M: morphine; Sevo: sevoflurane (%); Nad: noradrenaline (μg/kg/min); ICNB: intercostal nerve block; LA: local anesthesia

Case	Height (cm)	Weight (kg)	Anesthetic induction agents	Anesthetic maintenance agents	Regional anesthesia
1	162	63	Dex 2.9, R 0.04, Nad 0.03, MDZ 1 mg, F 25 μg	Dex 0.4, R 0.02-0.03, Nad 0.04-0.08, F 25 μg, M 3 mg	ICNB + LA
2	175	76	Dex 3.2, R 0.03, Nad 0.07, MDZ 1 mg, F 25 μg	Dex 0.4, R 0.03-0.05, Nad 0.03-0.04, MDZ 3 mg, F 75 μg	LA
3	171	57	Dex 4.2, R 0.1, Nad 0.015, MDZ 1.5 mg	Dex 0.7, R 0.05, Nad 0-0.07, MDZ 1 mg, F 100 μg	ICNB + LA
4	163	61	Dex 3.0, R 0.02, Nad 0.04, MDZ 1 mg, F 25 μg	Dex 0.4, R 0.02-0.04, Nad 0.03-0.04, MDZ 1 mg, F 75 μg, Sevo 1-2	LA
5	160	54	Dex 2.2, R 0.03, Nad 0.015, MDZ 0.5 mg	Dex 0.7, R 0.03-0.05, Nad 0-0.03, F 35 μg	ICNB + LA
6	154	44	Dex 1.8, R 0.03, Nad 0.05, MDZ 0.5 mg	Dex 0.9-1.8, R 0.03-0.04, Nad 0.03-0.05, MDZ 1.5 mg, F 50 μg	LA
7	181	97	Dex 3.0, R 0.02, Nad 0.04, MDZ 2 mg, F 50 μg	Dex 0.4-1.2, R 0.02-0.05, Nad 0.03-0.09, MDZ 1 mg, F 150 μg	LA
8	153	51	Dex 0.8, R 0.01, Nad 0.025, MDZ 1 mg, F 25 μg, K10 mg	Dex 0.8, R 0.02-0.1, Nad 0.01-0.025, MDZ 1 mg, F 75 μg, K 30 mg	ICNB + LA
9	165	46	Dex 3.5, R 0.03, Nad 0.1, MDZ 0.5 mg	Dex 1.7, R 0.02-0.03, Nad 0.1-0.45, MDZ 1.5 mg, F 85 μg	LA
10	151	55	Dex 0.7, R 0.015, MDZ 1.5 mg, F 25 μg, K 20 mg	Dex 0.7, R 0.03-0.21, MDZ 1 mg, F 75 μg, K 30 mg	LA

During anesthesia, patients with postoperative pericardial effusions were monitored by SpO_2_, electrocardiographic monitoring, and noninvasive blood pressure monitoring. In the case of shock, defined as cardiac tamponade, we added arterial pressure measurement. Central venous catheters were removed in all cases, so central venous pressure (CVP) could not be monitored. All patients were managed with spontaneous respiration.

## Results

**Figure 1:**
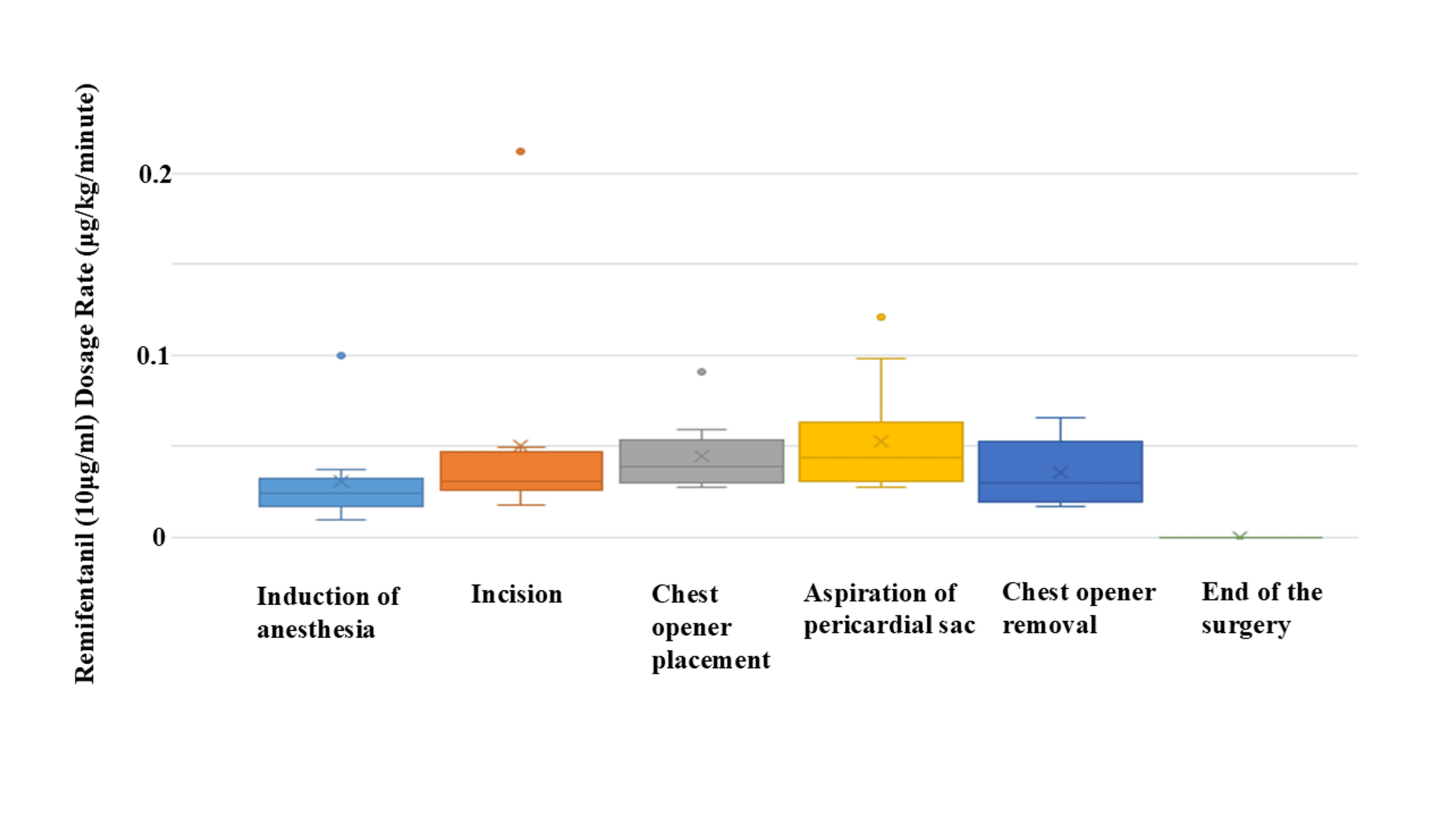
Actual diluted remifentanil (10 μg/mL) dosing rate (μg/mL/min) Remifentanil dosage changes for each case are shown. As the surgery progressed and became more invasive, the dosage increased. As the surgery came to a close and the invasiveness decreased, the dosage decreased. x: The mean value of 10 cases

In this study, we retrospectively investigated the anesthetic management of emergency pericardial fenestration with D-remi at a high infusion rate in critically ill patients. Table 2 shows the background of the patients. None of the patients had a history of glucose intolerance or dialysis. Two patients suffered from obvious shock and cardiac tamponade. Dyspnea was observed as a symptom of pericardial effusion in 8 out of 10 patients. Surgical aortic valve replacement (AVR) was the most common prior surgery, occurring in 5 of the 10 patients. Although there were six cases in which airway maintenance by mandibular elevation was required due to glossoptosis, the minimum intraoperative SpO_2_ was as high as 94% (92-100%), and none of the cases dropped below 92%. The drop in systolic blood pressure during induction was limited to only 29 mmHg (16-58 mmHg) (Table 3). In all cases, the emergency pericardial fenestration could be performed with stable hemodynamics throughout the procedure. The remifentanil dose in each case is shown in Figure 1. The dose of remifentanil increased with each surgical procedure: skin incision, use of a chest opener, and aspiration into the pericardial sac. The infusion rate of remifentanil decreased when the chest opener was removed. In one case, the dose of remifentanil was as high as 0.21 μg/kg/min due to severe pain during the skin incision, which was inferred from the patient’s grimace and movement.

**Table 2:** Patients background NYHA: cardiovascular disability assessment system of New York Heart Association; HTN: history of hypertension; CHD: history of coronary heart disease; PE: pericardial effusion; AVR: surgical aortic valve replacement; AAR: aortic arch replacement; MVP: mitral valvuloplasty; MVR: mitral valve replacement ＊1: Diagnosis by echocardiography;＊2: After percutaneous atrial septal defect device closure, the patient developed erosion and cardiac tamponade, and underwent emergency

Case	Age	Male/female	NYHA	Diagnosis	Symptoms	Prior surgery	Days since previous surgery	HTN	CHD	Anticoagulant medication	Antiplatelet medication
1	79	M	3	Postoperative PE	Fatigue, orthopnea	AVR	9	+	-	+	-
2	65	M	3	Postoperative PE	Delirium	AAR	10	-	-	-	-
3	57	M	4	Cardiac tamponade	Shock, dyspnea	AVR	9	-	-	-	-
4	75	M	3	Postoperative PE	Dyspnea, fatigue, loss of appetite	AVR	8	+	-	+	-
5	74	M	3	Postoperative PE	Dyspnea, walking, difficulty	AVR	6	+	-	+	-
6	73	F	3	Postoperative PE	Dyspnea, loss of appetite	AAR	23	-	-	-	-
7	55	M	2	Postoperative PE	Without symptoms＊1	MVP	19	-	-	-	-
8	72	F	2	Postoperative PE	Dyspnea	MVR	7	+	-	+	+
9	45	M	4	Cardiac tamponade	Shock, dyspnea, pale face	Thoracotomy and hemostasis＊2	11	-	-	+	-
10	72	M	2	Postoperative PE	Dyspnea	AVR	23	+	-	-	-

**Table 3:** Anesthesia management indexes HR: heart rate; sBP: systolic blood pressure; dBP: diastolic blood pressure; Nad: noradrenaline; Eph: ephedrine; Phl: phenylephrine

Case	Anesthesia time (min)	Operating time (min)	sBP/dBP on admission (mmHg)	HR on admission (beats/min)	Lowest sBP during operation (mmHg)	Decreased sBP with induction of anesthesia (mmHg)	Total opioid dose (μg)	Intraoperative lowest SpO_2_ (%)	Nad rate (μg/kg/min)	Total Nad dose (mg)	Total Eph dose (mg)	Total Phl dose (mg)	Infusion volume (mL)	ICU stay (days)
1	66	35	141/88	106	83	58	150	100	0.04	0.16	0	0.1	1100	2
2	104	65	105/72	84	86	19	270	92	0.07	0.3	0	0	1510	2
3	52	35	91/68	117	65	26	270	99	0.07	0.09	0	0.2	1140	1
4	64	42	124/76	142	101	23	200	94	0.04	0.17	0	0	600	1
5	95	47	153/96	85	99	54	175	93	0.03	0.08	0	0	1170	1
6	77	37	92/62	76	76	16	140	100	0.05	0.15	4	0	1150	1
7	86	48	133/88	96	81	52	400	94	0.09	0.45	0	0	1400	1
8	84	43	146/100	92	108	38	250	94	0.03	0.08	0	0	400	2
9	111	67	100/68	92	68	32	185	100	0.45	0.23	16	0.15	2050	2
10	92	48	137/80	84	121	16	300	93	0	0	0	0	400	1

## Discussion

In this article, we investigated 10 cases of emergency pericardial fenestration managed with MAC using D-remi. One case required a higher dose of remifentanil than the others - 0.21 μg/kg/min - due to severe pain at the skin incision. In a study comparing the efficacy of 2% lidocaine and 0.5% ropivacaine, Budharapu et al. measured the time to onset of effect in both groups. The onset of effect in the lidocaine group averaged 93 seconds, whereas the ropivacaine group took significantly longer, averaging 155 seconds [17]. In the present study, skin incisions were started before the effects of local anesthesia were felt, which may explain why one patient required a higher dose of remifentanil.

**Figure 2:**
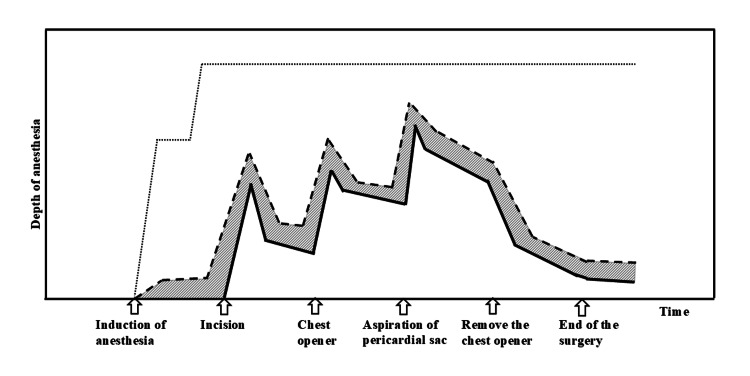
Conceptual diagram of target range of anesthesia by MAC for pericadial fenestration and actual remifentanil dosing rate The area between the dashed and solid lines represents the target range of anesthesia by MAC. The dotted line shows the theoretical depth of anesthesia if general anesthesia is administered. The patient’s spontaneous respiration may be compromised at the level above the dashed line, while the patient may feel pain at the level below the solid line. MAC: monitored anesthesia care

Ogumi et al. reported the usefulness of low-dose remifentanil for MAC in dental treatment, but the concentration of remifentanil used in their study was 100 μg/mL [18]. To the best of our knowledge, there are no reports of the use of remifentanil at 10 μg/mL in MAC. In our study, remifentanil was generally increased in dosage according to the timing of greater surgical invasiveness and decreased in dosage when surgical invasiveness was reduced (Figure 1). Based on these results, we developed a conceptual model of the target range of anesthetic depth required for MAC in pericardial fenestration. The area shown in Figure 2 would be the target area for anesthesia depth at the MAC in pericardial fenestration. If the MAC anesthetic depth is below the target area, the patient will have pain. On the other hand, if the depth of anesthesia is above the target area, the patient's spontaneous respiration and hemodynamic stability may be compromised. The dose of remifentanil must also be changed quickly to closely track the target anesthetic depth, which changes as the surgery progresses. Therefore, it is difficult to follow the target anesthesia area with the usual 100 µg/mL remifentanil and a low infusion rate.

**Figure 3:**
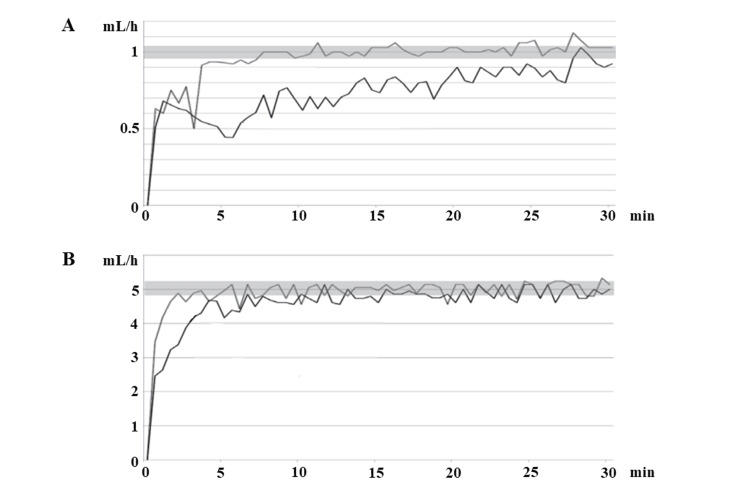
Start-up curve for administration flow rates of 1 mL/h (A) and 5 mL/h (B) The graph shows the actual ejection volume measured every second for a measurement period of 30 minutes. Graph A shows that, at a 1 mL/h output, it takes about 30 minutes for the actual output from the syringe pump to stabilize, while Graph B shows stabilization of a 5 mL/h output after only five minutes [2].

The average remifentanil used during the induction of anesthesia in the 10 cases was 0.031 μg/kg/min. If remifentanil is administered to a patient weighing 50 kg at a concentration of 100 μg/mL at 0.03 μg/kg/min, the infusion rate would be 0.9 mL/h. In contrast, a remifentanil concentration of 10 μg/mL at 0.03 μg/kg/min results in a dosing rate of 9 mL/h. The graph shown in Figure 3 is known as the start-up curve and illustrates the flow characteristics of the syringe pump. Yoshida et al. used an electronic balance and drove the syringe pump at 1 mL/h and 5 mL/h settings for two hours, measuring the actual volume ejected per second. Figure 3 shows the first 30 minutes of the two-hour period. At the 5 mL/h setting, the infusion rate quickly reached the set infusion rate and stabilized after just five minutes. On the other hand, when dosing was started at 1 mL/h, the infusion rate rose to about 60% of the set infusion rate in about five minutes, but from there, the infusion rate increased slowly, taking about 30 minutes after the start of dosing to stabilize at the set infusion rate of 1 mL/h [7]. Therefore, we believe that the faster the speed of drug administration by a syringe pump, the more accurate it is, and we believe that diluting remifentanil to 10 μg/mL and administering it at 10 mL/h is safer and more effective than administering it at 100 μg/mL at 1 mL/h. The time required for the actual output volume of the syringe pump to increase to the set infusion rate is shorter at higher dosing rates; thus, using D-remi allowed the actual dosing rate to be increased quickly in accordance with the expected timing of a large invasive event. Remifentanil is an opiate that tends to cause respiratory depression, but by diluting it, we were able to safely adjust the dosage rate. Combined with the higher infusion rate, we could quickly increase the dosage rate when the surgical stress was strong and immediately decrease it when the stress weakened. In our hospital, general anesthesia was often the anesthetic of choice for emergency pericardial fenestrations. However, as we began to transition to MAC, it gradually became accepted by cardiac surgeons to the point that they began to request MAC, especially in cases where there was a risk of circulatory collapse. General anesthesia is usually managed at RASS-5, while MAC aims for RASS-3 to -4. Less sedation is associated with less sympathetic inhibition, which is thought to have fewer adverse effects on cardiac circulation. The dose of analgesic medications is not limited by respiration, since the patient is under controlled respiration in general anesthesia. On the other hand, in MAC, the opioid dosage is titrated up to avoid loss of spontaneous respiration; as a result, intraoperative blood pressure may be preserved with a low opioid dosage. In addition, Carmona et al. listed ventilatory conditions as one of the key points of anesthesia induction for cardiac tamponade after cardiac surgery, noting that positive pressure ventilation has a significant impact on the cardiac tamponade patient’s hemodynamics, and maintaining spontaneous ventilation may be more tolerable than other modes of ventilation [19]. In cardiac tamponade with impaired venous return, negative pressure in the thoracic cavity, maintained by spontaneous breathing in MAC, may preserve preload and achieve more stable circulatory dynamics than under general anesthesia with positive pressure ventilation. Unfortunately, in this study, CVP was not measured, making it difficult to evaluate respiration and venous return. We believe that these multiple factors influence each other and that MAC management, as a so-called bundle, may have less effect on circulatory dynamics.

As mentioned earlier, comparative studies of general anesthesia and MAC have been performed for the anesthetic management of TAVI [20-22]. Villablanca et al. pointed out the advantages of MAC over general anesthesia in the anesthetic management of TAVI, including lower 30-day mortality and a reduced need for inotropic circulatory support [23]. Thiele et al. compared general anesthesia and MAC in the anesthetic management of TAVI and found that MAC produced similar outcomes in the primary efficacy endpoint compared to general anesthesia, suggesting that MAC can be safely applied to transcatheter AVR [24]. However, there are possible drawbacks to MAC in pericardial fenestration, such as patient movement, upper airway obstruction, and delayed response to circulatory collapse. In our case, spontaneous respiration never disappeared intraoperatively, but there were instances in which body movements occurred during strong surgical stress, such as during traction of the ribs or stimulation of the pleura. In 6 out of 10 cases, the mandible had to be manually raised to secure the airway due to glossoptosis, but the frequency and duration of these instances could not be evaluated due to a lack of records. However, there were no cases in which SpO_2_ fell below 92% intraoperatively. It is difficult to successfully match the depth of anesthesia and the infusion rate of analgesics to increases or decreases in surgical invasiveness. However, using remifentanil, which has a short half-life, as the primary analgesic and diluting it to achieve a higher infusion rate may help with this issue.

## Conclusions

We investigated anesthetic management with MAC in patients who underwent pericardial fenestration for postoperative pericardial effusion. The use of more D-remi than usual allowed us to set the syringe pump to a higher infusion rate. This increased the responsiveness of the syringe pump to changes in the set infusion rate. The improved responsiveness of syringe pumps through the use of D-remi may be advantageous in situations where the infusion rate of anesthetic needs to be adjusted frequently and quickly. Since this is a small case study, we view our results cautiously and hope that further investigation of the usefulness of D-remi in MAC, including large-scale randomized studies, will be conducted in the future.
